# Implementing a digital infrastructure for the lab using a central laboratory server and the SiLA2 communication standard

**DOI:** 10.1002/elsc.202000053

**Published:** 2020-12-09

**Authors:** Marc Porr, Ferdinand Lange, Daniel Marquard, Laura Niemeyer, Patrick Lindner, Thomas Scheper, Sascha Beutel

**Affiliations:** ^1^ Institute of Technical Chemistry Leibniz University Hannover Hannover Germany

**Keywords:** internet of things, laboratory device communication, laboratory digitization, laboratory network, SiLA2

## Abstract

In this report, a fully integrated solution for laboratory digitization is presented. The approach presents a flexible and complete integration method for the digitally assisted workflow. The worker in the laboratory performs procedures in direct interaction with the digitized infrastructure that guides through the process and aids while performing tasks. The digital transformation of the laboratory starts with standardized integration of both new and “smart” lab devices, as well as legacy devices through a hardware gateway module. The open source Standardization in Lab Automation 2 standard is used for device communication. A central lab server channels all device communication and keeps a database record of every measurement, task and result generated or used in the lab. It acts as a central entry point for process management. This backbone enables a process control system to guide the worker through the lab process and provide additional assistance, like results of automated calculations or safety information. The description of the infrastructure and architecture is followed by a practical example on how to implement a digitized workflow. This approach is highly useful for – but not limited to – the biotechnological laboratory and has the potential to increase productivity in both industry and research for example by enabling automated documentation.

**Abbreviations**
AnIML: analytical information markup language
API: application programming interface
CIL: common intermediate language
FAIR: findability, accessibility, interoperability, reusability
gRPC: google remote procedure call
(G)UI: (graphical) user interface
ID: (unique) identifier
IPC: inter process communication
JSON: javascript object notation
NFDI: nationale forschungsdateninfrastruktur
PCR: polymerase chain reaction
POSIX: portable operating system interface
Proxmox VE: proxmox virtual environment
QR code: quick response code
RS232: recommended standard 232
REST: representational state transfer
RPC: remote procedure call
SiLA2: standardization in lab automation 2
SSH: secure shell
TCP/IP: transmission control protocol/internet protocol
USB: universal serial bus
(W)LAN: (wireless) local area network
XML: extensible markup language

## 1 INTRODUCTION

In this article, a concept for digital integration of a biotechnological laboratory is presented. The main goals are centralized and automatic acquisition of data and metadata, enabling assistant technologies for the researcher and automated documentation. In contrast to decentralized approaches [1, 2], this method is focused on a centralized control‐ and data‐management system that interacts with human inputs and orchestrates procedures based on the results and outcomes of previous steps. Most digitization concepts available focus on highly automated laboratories [3, 4, 5] with processes that require only a small amount of human‐machine interaction [6]. This creates automated and smart “digitized islands” in the laboratory that are surrounded by an ocean of dumb lab equipment and devices. Often these islands are not standing separate but other devices and manual procedures are needed for additional steps. Even though this user interaction problem was named by Frey in 2004 [7], interaction of the digitized lab with humans is still a daunting task today. Especially in the biotechnological lab, where complex protocols are carried out and a lot of additional information has to be processed by the researcher, a method for adding digital support seems promising.

In this approach, all devices and resources can be accessed in a central place. The laboratory server guaranties a full record of measurements, results and events. This enables possibilities for certification – for example GxP (Good Manufacturing/Laboratory Practice) compliance [3]. Also implementing FAIR (Findable, Accessible, Interoperable, Reusable) data principles [8], which are for example required by the NFDI (Nationale Forschungsdateninfrastruktur), is possible.

**PRACTICAL APPLICATION**

The presented architecture can be used to transform a traditional laboratory into a digitized one. It focuses on the interaction with the laboratory worker in processes that cannot – or should not – be automated. Procedures are defined in advance and are carried out in a structured way that ensures a correct workflow. The architecture centralizes information and laboratory device communication in a server, which also hosts a database that stores all data generated in the laboratory. The focus lies on small research or analysis laboratories where procedures are not highly automated, but where data quality and productivity can significantly benefit from digital assistance. This opens up possibilities of interactive workflow guidance for the lab worker, automated result documentation and an improved control over the lab processes. Workers can spend more time on productive tasks when calculations, data analysis and documentation are carried out fully automated in the background.

For good maintainability, a micro‐service based architecture was chosen. The approach focuses on standardized communication of all components. This enables good horizontal scaling, which is important in digital transformation of existing infrastructures [9]. One central Representational State Transfer (REST) Application Programming Interface (API) is used for operation procedure orchestration and data requests. All devices are connected using the Standard in Lab Automation 2 (SiLA2) standard for laboratory device communication^1^. The usage of one single standard highly increases the flexibility and usability of the setup [10]. The first stable version of the SiLA2 standard was released in 2019 and several implementations in different programming languages are available today^2^. The “race for the lab communication standard” is on [11] and nobody can predict which standard will be widely adopted in the future. However, with its open source concept and good documentation, SiLA2 offers some significant advantages for the developer that aims on integrating existing devices without a common communication principle. For these kind of devices, a “translator” for SiLA2 must be used [5]. This, at first sight, might look like only shifting implementation efforts. However, as no common standard for lab device integration exists, the focus on one standard wants to empathize the need for standardization and flawless integration. Furthermore, from a software architect's point of view, the separation of concerns is an important pattern in complex systems. When using a standard, the laboratory server does not need to know about all the different protocols for all devices. It can be kept simple in both design and implementation, which is important for good scalability and maintainability. This approach divides the big integration problem into small separate tasks. All translators share a common structure and form a swarm of micro‐services that are managed from a central web‐interface of the laboratory server. In addition, deployment of these services is automated by a central publish‐system. Both the management and publish system, as well as a guide on how to write SILA2 translators as micro‐services, were published by the authors before [12] and are available under an open‐source license.

Devices that cannot be connected directly to the laboratory network, are integrated using a gateway module. This was described in detail by the authors before [12] and all necessary information and software are available under open licenses.

The flexible nature of the system and the central REST interface of the laboratory server enable easy connecting of different process management tools. Using an appropriate middleware, an electronic lab notebook or any other form of data structuring or data analysis solution can easily be connected to the lab server as well.

The communication of the laboratory system with the researcher is modeled using a standard gateway to all possible user interface devices (UI‐devices). For example, these can be head‐mounted displays (“smart‐glasses”), tablets, etc. Due to its complexity, the user‐interface‐system and the process management tool co‐implemented with this system cannot be described in detail in this article. However, the interested reader can find the details in the supplementary data.

Traditionally, a researcher needs to follow a printed standard operating procedure and must document all steps by hand. Additionally, interpreting, calculating and archiving of result data needs to be carried out manually afterward. Using the presented architecture, these tasks can be automated and standardized. This is possible due to the central data storage and fully flexible availability of data.

This increases data quality [5, 6, 11] as human errors or mistakes in copying notes are much less likely. The automated data management takes the burden of result documentation from the researcher and leaves more time for performing qualified tasks. This may help to overcome the lack of skilled labor currently experienced in the fields of laboratories and research [3, 4, 6, 11] – especially in the biotechnological sector.

## 2 MATERIALS AND METHODS

This approach relies on a dependable infrastructure to ensure fast and secure communication of the distributed systems. Wherever possible existing technologies were used. Today's technology is quite capable of performing all relevant tasks in a digitized lab. The current hype in home automation and the “internet of things” shows what can be achieved when manufacturers agree on communication standards [13]. However, such industry standards are not yet present in the laboratory world. Therefore, some effort has to be made to enable devices from different manufacturers to exchange data and commands [5].

### 2.1 Hardware integration

**FIGURE 1:**
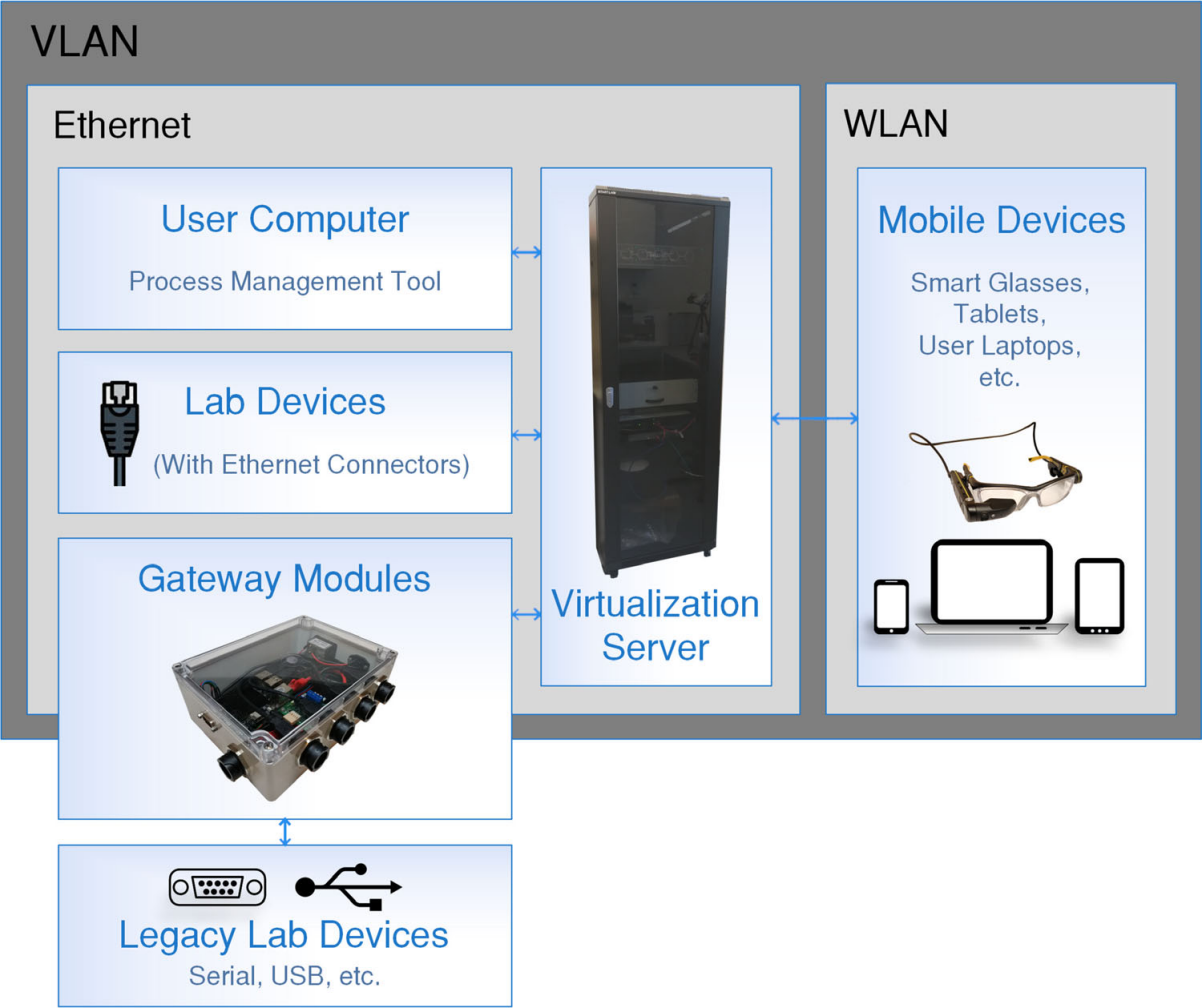
Overview of the hardware used in the digitized laboratory. All components are connected to the same VLAN (Virtual Local Area Network) either with wire‐based Ethernet connections or to WLAN. Legacy devices are connected to the network using hardware gateway modules. With this network architecture, the flawless communication of all devices is ensured

Figure 1 shows an overview of the hardware used for the digital network. For communication of digital components Transmission Control Protocol/Internet Protocol based protocols are widely used and easily scalable. Thus, an Ethernet‐based local area network with DHCP (Dynamic Host Configuration Protocol) forms an adequate base for lab digitization. Wherever possible wire‐based connections were used due to their reliability. User interface devices, like wearables or handheld devices are connected via WLAN (Wireless Local Area Network) access points. User computers, which run protocols and interact with the lab server, are connected to Ethernet ports in the lab or via WLAN.

Lab devices with an Ethernet port can easily connect to the lab network, as no further hardware is needed. Older or simpler devices do not have Ethernet or WLAN capabilities. These devices can usually be connected to a controller or computer by a serial‐ or Universal Serial Bus (USB)‐connection. These connection methods are not easily scalable, not sharable and cable lengths are limited. This means a lab server would have to be connected to every of those devices directly. As the server will usually not be in the same physical location as the devices but in a dedicated server room, connecting legacy devices is a great challenge. A gateway‐module was used to integrate such devices into the digitized lab network. The building and programming of this gateway module was described in detail by the authors before [12] and all plans and software are available under an open‐source or open‐hardware license. The module is part of the laboratory network and connects to the lab device via USB or serial/Recommended Standard 232. Communication from the module to the device is done in whatever protocol is required by the specific device. One module can connect multiple lab devices, which can be desirable if they are placed close together.

### 2.2 Data transfer and protocols for device integration

For data transfer, standardized network protocols were used. The lab server offers all functionality via a single RESTful^3^ Hypertext Transfer Protocol web service. All devices are controlled with the SiLA2 protocol.

SiLA2 focuses on consistent communication standards for all devices. It is based on the Google Remote Procedure Call Protocol. All device commands and properties are logically organized in “features.” One device can implement several features, which can interact and modify each other if necessary. For example, a magnetic stirrer would implement a “heating,” a “stirring,” and the mandatory “SiLA2” standard feature. The service, that hosts features and presents them in the network, is called the SiLA2 server. A SiLA2 client can establish a network connection to the server, use the standard feature to obtain documentation on all implemented features and initiate command execution on the device according to this in‐place self‐documentation.

So far only very few lab devices offer their functionality in form of a SiLA2 server off the shelf. This raises the need for “translators” or gateways between SiLA2 and the vendor‐specific protocols. For legacy devices that are connected via the hardware gateway module, the SiLA2 servers for these devices are running on the embedded computer of the module. For every other device, one virtual machine hosts the specific SiLA2 server.

### 2.3 Virtualization server

To minimize the demand for hardware and to ease maintenance and scalability a Proxmox Virtual Environment virtualization server is used [14]. Server applications and virtual gateways to lab devices all run as virtual machines on this central virtualization server. One laboratory needs at least one machine for hosting the lab server, one per network‐connected device and some more for infrastructure requirements (i.e. controller for WLAN access points, data server(s), etc.).

## 3 RESULTS AND DISCUSSION

**FIGURE 2:**
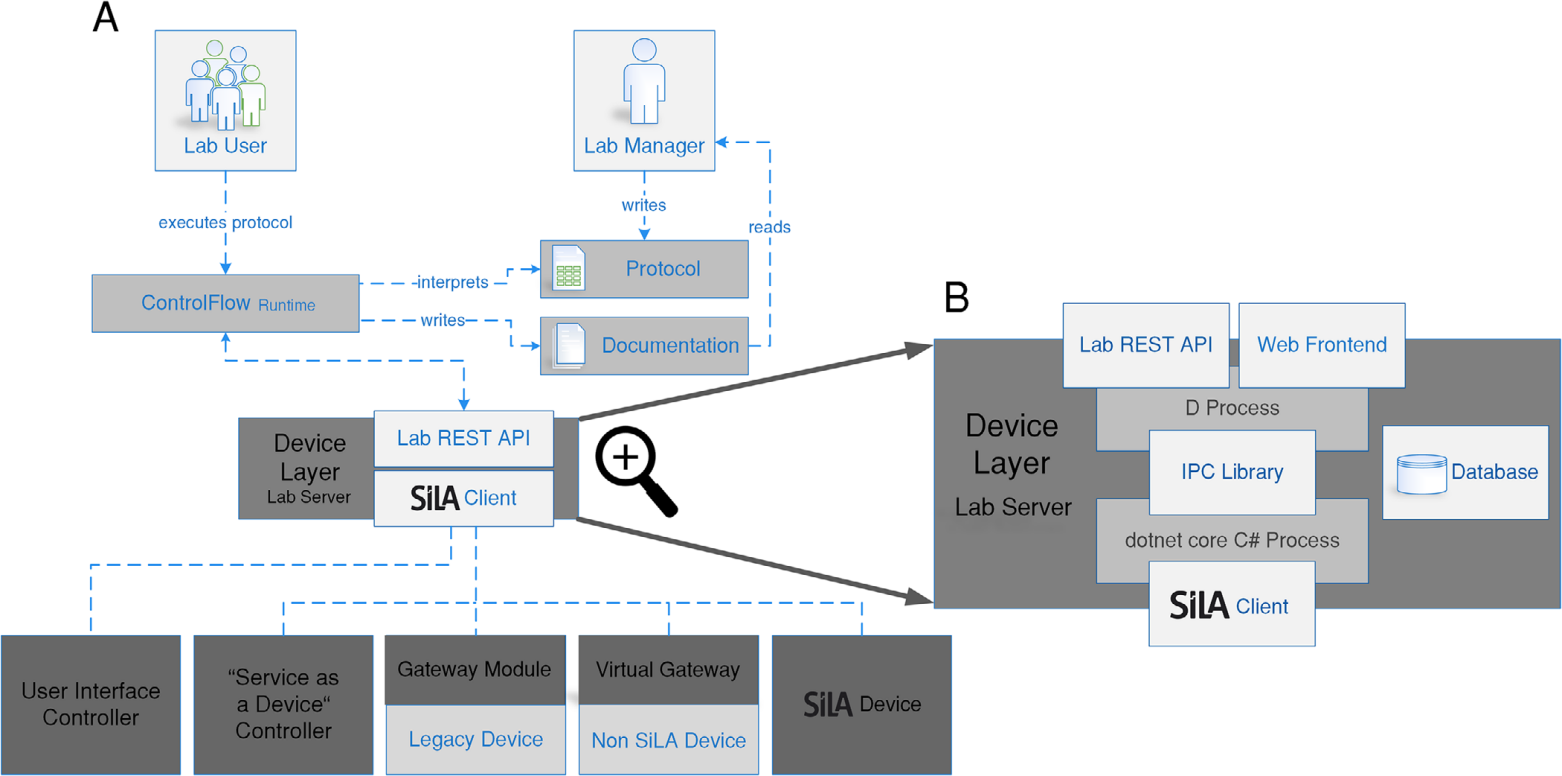
The digitized laboratory is based on a central lab server (DeviceLayer). The DeviceLayer implements a generic SiLA2 client that connects all laboratory devices. This is done either directly, via a virtual gateway or using a hardware gateway module. A virtual user interface controller generalizes interaction with UI‐devices. Services or resources are implemented as SiLA2 servers and are connected the same way as lab devices. The DeviceLayer also implements a web frontend for administration and a REST API for interacting with the laboratory. All data from the lab is stored in a database that is also running on the same machine as the two DeviceLayer processes

Following a micro‐service based strategy the system is implemented as several small components that communicate and interact with strictly defined and standardized interfaces or APIs. In Figure 2A an overview of all soft‐ and hardware components and their interaction with the human lab manager and researcher is given. The complete system is implemented using free software and the key parts of it were made publically available under appropriate open‐source licenses (see Table 1).

**TABLE 1:** Summary of all tools and libraries used for the presented system including references to documentation and download sources

Element	Tools and libraries used	References
Virtualization server	Proxmox Virtual Environment version: 6.1	https://www.proxmox.com/en/proxmox-ve
SiLA2 servers for: UI controller “Service as a Device” controller Gateway modules Virtual gateways	sila_tecan SiLA2 Implementation (C#) Git commit used:@45b977b6 Publish‐System (bash) Git commit used: @0c7a5778	https://gitlab.com/SiLA2/vendors/sila_tecan https://gitlab.uni-hannover.de/tci-gateway-module/gateway-publish Detailed descriptions for developing and deploying in [12]
Gateway modules	Based on Embedded Computer: Hardkernel ODroid C2 Purchased from Hardkernel Co. Ltd. In December 2019 Ubuntu Linux Version 3.16 from ODroid‐wiki (based on Ubuntu 18.04.3, update‐state Mai 2020) sila_tecan SiLA2 Implementation (C#), ported to dotnet core for embedded applications (see Branch “tci‐gwm”) Git commit used: @45b977b6 gRPC (forked to work with embedded processor architectures) Git commit used: @0d417d55 based on: @c564d28f in official repository	https://wiki.odroid.com/odroid-c2/odroid-c2 https://ubuntu.com/ https://gitlab.com/SiLA2/vendors/sila_tecan/-/tree/tci-gwm https://github.com/grpc/grpc https://gitlab.uni-hannover.de/tci-gateway-module/grpc Detailed descriptions for building, installing, programming and running in [12]
Virtual gateways	Arch Linux Linux kernel version: 5.0‐5.7, tested with updated rolling system in Mai 2020	https://www.archlinux.de/
DeviceLayer lab server	Arch Linux Linux kernel version: 5.0‐5.7, tested with updated rolling system in Mai 2020	https://www.archlinux.de/
Generic SiLA2 client in DeviceLayer	DynamicClient from sila_tecan SiLA2 Implementation (C#) Git commit used: @45b977b6	https://gitlab.com/SiLA2/vendors/sila_tecan
IPC library in DeviceLayer	POSIX Message Queues (Utilized In C# and D) Tested with version from Linux 5.0‐5.7	https://www.man7.org/linux/man-pages/man7/mq_overview.7.html
REST server and web‐frontend in DeviceLayer	vibe.d REST and web application framework (D) D, dmd/phobos version: 2.089 vibe.d version: 0.8.6	https://vibed.org/
Database in DeviceLayer	mongoDB tested with version: 4.0.6	https://www.mongodb.com/
ControlFlow library and runtime	curl and curl D library (D) Library D, dmd/phobos version: 2.089 Tested with curl version: 7.67 imgui GUI library (C++ library invoked from D) commit used: @3bde3750 (v1.75 WIP + docking)	https://curl.haxx.se/ https://dlang.org/phobos/std_net_curl.html https://github.com/ocornut/imgui

All components are either based on open‐source software or have been previously described and released by the authors under an open‐hardware or open‐source license [12]

All devices are connected to a central laboratory server (DeviceLayer) using the SiLA2 protocol. For devices that do not offer SiLA2 compatibility directly, gateway is in place. This can be either a virtual machine or a physical gateway module. User interface devices are abstracted by a generic UI‐device gateway.^4^ The DeviceLayer offers a uniform interface for any kind of software to connect to the laboratory. A process control system can use this interface to issue commands on lab devices, collect measurement results and orchestrate steps accordingly. A process management tool (“ControlFlow Runtime”), which is highly focused on user‐friendly interaction of the digitized lab with the researcher, was developed in this approach and is described in detail in the supplementary materials.

### 3.1 Laboratory server (DeviceLayer)

The central component of the digitized lab is the DeviceLayer laboratory server. One instance of the DeviceLayer forms the virtual representation of “a laboratory” in means of all devices and resources used together. Figure 2B shows the implementation principles of the DeviceLayer. All communication is channeled through this component to ensure full control and data integrity. A database stores every event that occurs in the lab. Furthermore, the Device Layer acts as a central interface for administration. A lab manager can monitor the status of all connected devices and their gateways with a single web interface. The source code for this web interface was described before [12] and is publicly available under an open‐source license.

The DeviceLayer communicates with all devices and service‐providers using SiLA2 and presents the entry point for all user‐side software by hosting a REST API in the network that wraps up all device functions.

The DeviceLayer software is implemented as two separate processes that communicate using an inter process communication mechanism based on Portable Operating System Interface message queues. One part of the DeviceLayer is written in dotnet core C#. It implements a generic SiLA2 client. For that, the “DynamicClient” from the open‐source sila_tecan library is used. All devices are connected to the DeviceLayer and one instance of that client is generated for every lab device. The other part of the DeviceLayer is written in D and consists of a database API, the RESTful Hypertext Transfer Protocol web API and a web frontend for laboratory administration purposes. For implementing these three components, the open source vibe.d library is used. An open source NoSQL database (mongo DB) is running on the same virtual machine to store all data of every command executed and every result generated in the lab. The database API models the connection for the other components of the DeviceLayer to use. The REST‐API offers all device functionality from the whole lab in one place to interact with for a process management tool or any other client. It can also be used to access data from the database.

This API acts as a central entry point to the lab and offers a consistent way of interacting with all kinds of devices and services. When a command or property is requested for a specific device, the availability of the desired device is checked. If the device accepts commands, the SiLA2 call is generated and passed through to the device's SiLA2 server. The command's results are then available for further processing or archiving.

Every command execution is given a unique identifier (ID) by the API and all final or intermediate results are stored in the database. They remain accessible using this specific ID, even when the devices used to carry out the operation seize to work.

**FIGURE 3:**
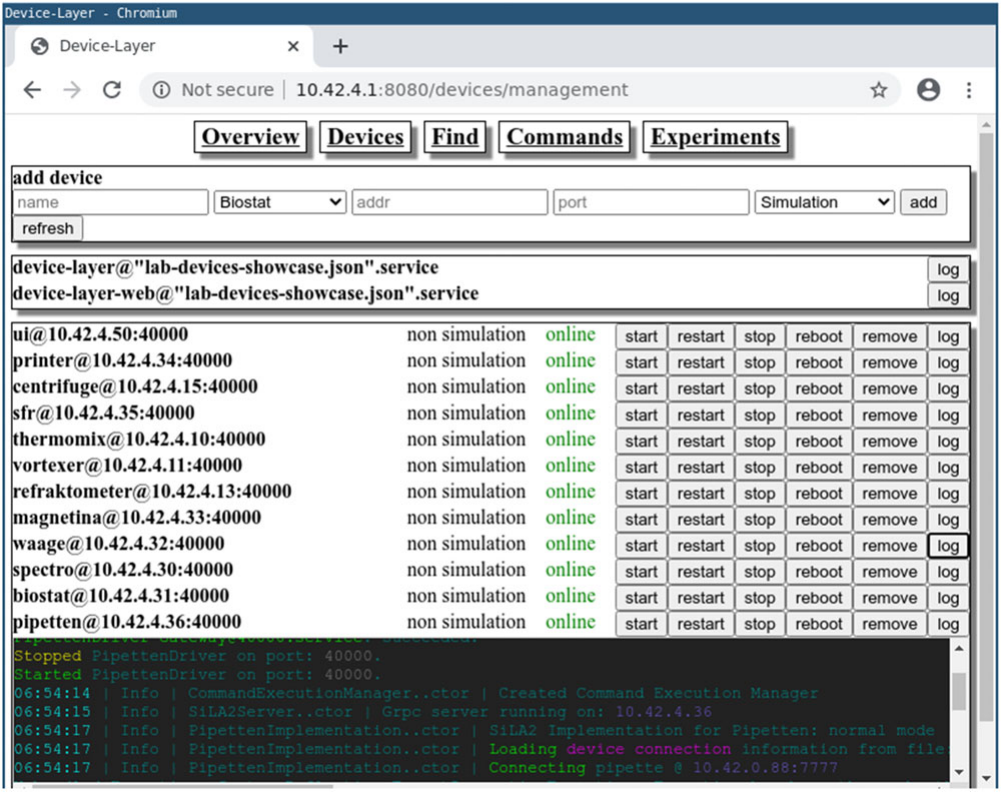
Screenshot of the web frontend hosted by the DeviceLayer

The DeviceLayer also hosts a web interface (see Figure 3) that can be used by the lab manager to administrate all laboratory devices. The devices states can be monitored and gateways can be remote controlled. In addition, logs from all SiLA2 servers in the lab are available. SiLA2 devices can be attached to or removed from the lab during runtime of the DeviceLayer.

### 3.2 Lab device integration with SiLA2

The SiLA2 Protocol is used for all device communication from the DeviceLayer to lab devices. This means, every device is hosting a SiLA2 server that presents all device functionality on the network. This ensures a streamlined design of the DeviceLayer and encapsulates device integration tasks in small subunits that do not interact with each other. In case several devices of the same type are in use or several devices of the same manufacturer “speak the same language,” these units can be duplicated and scaled easily in a horizontal way.

A generic user interface controller, which is also implemented as a SiLA2 server, generalizes interaction with UI‐devices (like tablets or smart‐glasses). Services or resources are wrapped by SiLA2 servers and are connected the same way as lab devices.

#### 3.2.1 SiLA2 virtual gateways and gateway modules

Today most devices do not come with a standardized interface and almost no devices offer SiLA2 capabilities. This raises the need for “translators” or gateways from manufacturer/device‐specific protocols to SiLA2. Depending on each device‐type, either a virtual gateway or a gateway module is used. Every gateway is a standalone application that hosts a SiLA2 server and transforms SiLA2 commands to device specific calls. These are transported depending on the hardware interface of the device. Many new devices offer common interfaces to their devices, such as REST, Extensible Markup Language‐ Remote Procedure Call or JavaScript Object Notation. Nevertheless, often these services fail in following the chosen standard fully and some extra work has to be done to circumvent this. These devices can be integrated in the lab network directly and a translator gateway can interact with these devices via Transmission Control Protocol/Internet Protocol‐communication. Thus, a virtual machine on the virtualization server can run one or more gateways for Ethernet‐capable devices. In the solution presented here, for reasons of conformity and scalability, every virtual gateway runs in its own virtual machine.

For legacy devices that only come with a serial‐ or USB‐connector, hardware gateway modules are used. These modules essentially consist of an embedded computer that is connected to the lab device and runs the SiLA2 for that device. The module itself is connected to the lab network via Ethernet and thus enables the flexible integration of older or simpler devices.

All gateways are implemented in dotnet core C# using the open source sila_tecan library. sila_tecan was ported to dotnet core, which allows using the same software on the gateway module and virtual gateways. sila_tecan and also the dotnet core port are available under an open‐source license. The usage of the ported variant and also the modifications necessary to use it on the embedded hardware of the gateway module were described by the authors in detail [12]. The gateway modules run Ubuntu/Debian Linux systems, whereas the virtual gateways are running Arch Linux systems. For devices that need manufacturer software that runs uniquely on Windows, the platform independent dotnet core runtime can be used in the same way. The gateway module was released by the authors of this article under an open source/open hardware license [12] and SiLA2 driver development with dotnet core C# using the sila_tecan library was also described in a step‐by‐step tutorial [12].

#### 3.2.2 Development and maintenance of SiLA2 servers in a distributed system

**FIGURE 4:**
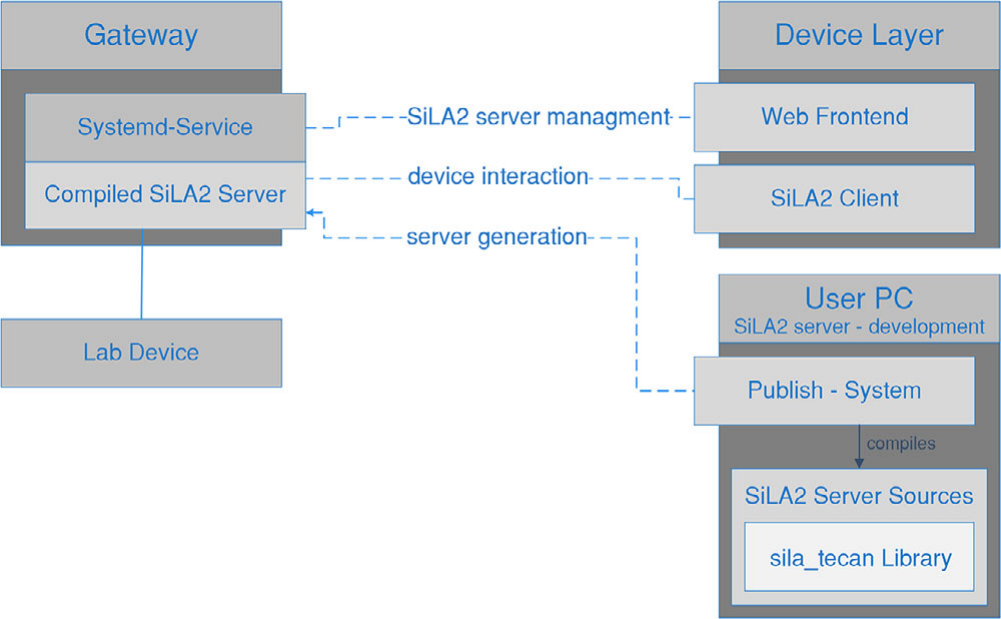
Workflow for SiLA2 server development and usage of the publish system and method of remote control of the SiLA2 servers from the DeviceLayer administration web frontend

To simplify the development, testing and deployment workflow, a publish system was implemented. Figure 4 visualizes the workflow of the publish system. The developer implements the SiLA2 server functionality on a desktop computer using dotnet core C# and the sila_tecan library. For performance reasons, the publish system precompiles the dotnet core source code to Common Intermediate Language code that is platform independent and can be executed by a dotnet core runtime on the gateway modules or the virtual gateways. The publish system then transfers this software package via Secure Shell to the target gateway and registers a systemd/systemcontrol service for remote controlling the SiLA2 server. This service is controlled by the lab manager via the DeviceLayer's web frontend. The publish‐system was described in detail before [12] and is available under an open‐source license. It is based on bash‐scripts, which were extended for this application by a method for a configuration‐file based “batch‐deployment,” that can update or reinstall the whole lab at once.

#### 3.2.3 Services and resources as devices

Following the “divide and conquer” principle, non‐device services were also wrapped in SiLA2 servers. These are integrated into the digitized laboratory via a virtual gateway in the same way, as actual devices would be. This approach generalizes knowledge and keeps the protocols thin. For example, a complex mathematical computation, such as a process modeling based parameter estimation, becomes a “device” that has a SiLA2 interface. It can be used from within the protocol script via the Lab REST API in the same way as any other lab device.

In a similar fashion, other services, such as automated image analysis tools or an AniML (Analytical Information Markup Language) generator can be integrated. In addition, a resource planner or scheduler can be added. When there are multiple devices of the same type in the laboratory, a “meta‐device” for scheduling would implement the same SiLA2 interface as the devices themselves. The protocol would only interact with that scheduler and the task would automatically be performed on the first device from the pool becoming available.

### 3.3 Practical example for the setup and execution of a digitized workflow

To increase understanding, an example workflow for water analysis is now discussed. The focus lies on highlighting the principle steps that need to be taken to encounter a digital transformation. Due to reasons of complexity, this cannot be a detailed step‐by‐step tutorial. However, the systematics for SiLA2 gateways and its central administration, the implementation of SiLA2 servers, the usage of the publish system and the underlying principles for system and software design are all available under open‐source licenses. They were described in detail (including step‐by‐step bash command instructions and extensive source code documentation) in an earlier publication [12].

#### 3.3.1 Technical requirements and protocol description

As a starting point it is assumed, that the hardware and infrastructure is set up as described in the Materials and Methods section. A local area network that can connect all components is in place. A laboratory server running the DeviceLayer is accessible in this network. The DeviceLayer is the central entry point for administration of all lab devices (web‐frontend), process management (REST‐API) and data storage (database). A hardware gateway module has been build and installed following the instructions in [12].

**FIGURE 5:**
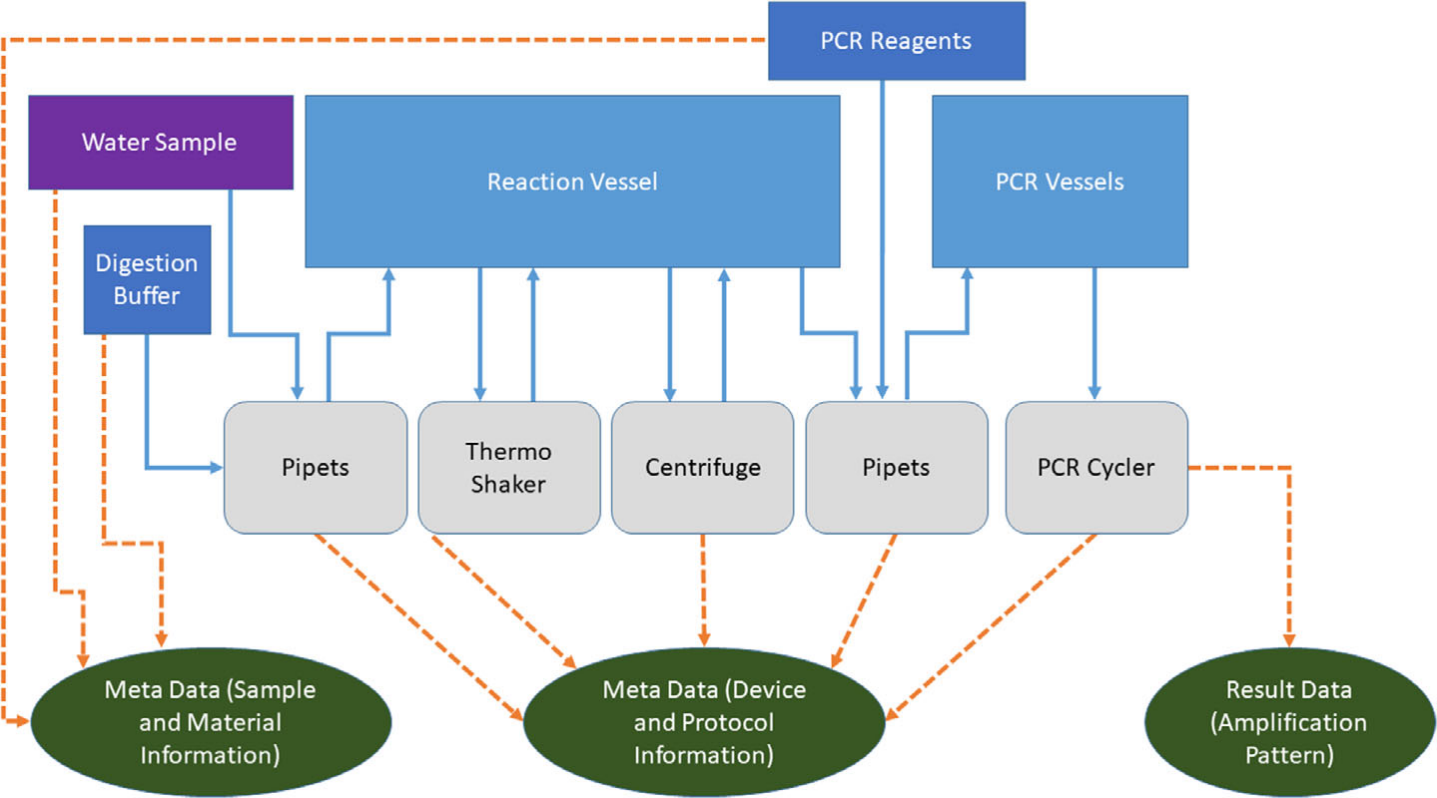
Schema of the exemplary water analysis workflow. The sample is represented by the purple box, all inventoried materials by the dark blue boxes. Light blue boxes: reaction vessels; Grey boxes: lab devices. Data generated by the process is displayed in the green cycles. Solid blue arrows: physical transfer of materials or vessels; Dashed orange arrows: data generated in specific locations

The schematics of the water analysis workflow are depicted in Figure 5. A given water sample should be analyzed for microbial contamination. For that, a defined volume of the sample is first transferred into a digestion buffer. The digestion is carried out in a combined heating and shaking device (thermo‐shaker). After the digestion, the cell debris is separated by centrifugation and the supernatant is used for a Polymerase Chain Reaction (PCR) based detection of microbial genetic material.

For this rather simple workflow, four lab devices are needed: pipettes, thermo‐shaker, centrifuge and PCR cycler. It is assumed, that all devices offer some kind of digital interface to allow remote control and data aggregation. The pipettes are connectable to a WLAN network and are controlled by a JavaScript Object Notation‐Remote Procedure Call protocol. The thermo‐shaker has a Recommended Standard 232 serial interface and can be controlled by a simple serial protocol. The centrifuge has a USB connector, which is mapped internally to a USB‐serial converter. Thus, this device is also controlled by a serial protocol. The PCR cycler has an Ethernet connector and runs a REST server that offers endpoints to control the device. This example situation reassembles a common starting point in lab digitization.

Additionally smart‐glasses offer the possibility to guide the user through the process and provide additional information. The existence of an inventory system is assumed, that is used for chemical registration. All chemical containers are registered with this system and are identifiable via a ID that is printed on the containers as a Quick Response code. The inventory system offers a REST‐API to access all information regarding a special ID.

#### 3.3.2 Device integration and workflow setup

To connect the devices needed for this workflow to the laboratory server, they first need to be placed in the network. For the pipettes and the smart‐glasses, this means connecting them to a WLAN access point in the laboratory network. The PCR cycler is plugged into the same network using an Ethernet cable directly. The centrifuge (USB‐connector) and thermo‐shaker (serial‐connector) are plugged into the gateway module which itself is connected to the lab network using its Ethernet port.

After these physical requirements are met, all devices need to be linked to the laboratory server. This means that for every device a SiLA2 server needs to be running in the network that translates SiLA2 calls to the device specific protocol. For the pipettes and the PCR cycler virtual machines on the virtualization server are set up to run those servers. For the centrifuge and thermo‐shaker, the SiLA2 servers are to be run on the gateway module. The developer now needs to implement all SiLA2 servers following the micro‐service template pattern that is used by the publish system. This essentially means describing and documenting the SiLA2 interface and wrapping up the device specific calls described in the devices manual into the SiLA2 structure. The implementation of a SiLA2 server using the sila_tecan library and dotnet core C# is documented in detail in [12] and in the “SampleServer” in the sila_tecan repository. Also the special requirements (using a Google Remote Procedure Call implementation modified for embedded processor architectures) outlined in [12] needs to be taken into account when developing servers for the gateway module.

When the SiLA2 servers are available and were compiled and deployed to the target machines (refer to [12] for detailed instructions) they can be added to the DeviceLayer using the web‐frontend.

For the user interface on the smart‐glasses, the generic UI SILA2 server abstracts the communication to the DeviceLayer.^5^ Following the “Services as Devices” pattern, the inventory application is wrapped in a SiLA2 server that also runs on a virtual machine. If the results of the experiment should be available according to the FAIR data principles [8], a generator, that takes all experimental data and metadata and generates an AnIML file, which it uploads to a publically available repository, can be integrated in the same way. This enables the usage of the system for example in context of the NFDI. When a repository for research data are available (either locally or in the internet), these standardized data can be uploaded to it automatically by the AnIML‐Generator or a specialized micro‐service.

After adding the SiLA2 servers to the device list in the DeviceLayer, all necessary functionality of the lab can be accessed using its REST API. To set up a workflow, a process management tool needs to be connected to this API. For this example the ControlFlow library is used, that is described in the supplementary information. Workflows are described in a script‐like format and are made up of steps and transitions between them.

**FIGURE 6:**
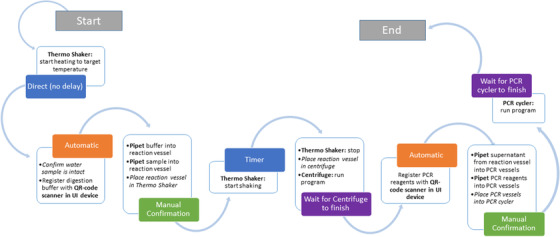
Simplified overview of the steps and transitions necessary for the water analysis example workflow. White boxes represent step content. Bold font: a device is utilized; Italic font: manual tasks with no direct digital interaction. The colored boxes attached to the steps represent the transition conditions that need to be fulfilled for leaving a step. Blue: transitions with no direct interaction with the lab or the researcher (timers or direct transitions); Orange: transitions, which occur as soon as the last task inside the step is performed; Green: transitions require the manual confirmation of the researcher (pushing a button or using a speech command on a UI device); Purple: transitions that wait for a device command to finish

For this example workflow, the simplified protocol layout is shown in Figure 6. The corresponding ControlFlow script can be found in the supplementary information. The script is compiled to a ControlFlow Runtime, which can be executed by the lab user on any computer connected to the lab network.

When starting a run of this ControlFlow Runtime a unique experiment ID is automatically created in the DeviceLayer database. All experimental data generated during this run is automatically linked with this ID. This creates structural context of result data from different devices and links this with additional metadata about the experimental setup. For accessing the DeviceLayer database, the REST‐API can be used by any data analysis tool (either directly or via a connecting middleware). For quick database queries, the DeviceManager web frontend of the DeviceLayer offers a graphical tool for viewing experimental data.

#### 3.3.3 Execution of the digitized workflow

After all necessary devices and the workflow script for the process control system are in place, the new digitized workflow can be started. During the process, many different kinds of information are available and are presented by the system at different levels of abstraction.

The lab user is presented on the smart‐glasses with screens that display short information snippets about the current step of the protocol or context sensitive information. During the pipetting steps the materials to use are shown together with relevant safety information, whereas during the denaturation or centrifugation step, the remaining runtime of the device is displayed. The smart‐glasses are also used to scan the Quick Response‐codes generated by the inventory system. The contents of these codes are fed into the “virtual” inventory device. This enables runtime checking for correctly used chemicals and expiration dates. It also enables the inventory system to track the use of all registered materials automatically.

The ControlFlow executable presents the user with a graphical user interface that allows process coordination (jumping between steps, re‐running steps, etc.) and shows the communication between devices and lab server in a condensed form. The lab manager can use the DeviceLayer web‐frontend to view the logs from all SiLA2 servers in the laboratory in one place. This can be used for validating processes or troubleshooting and testing new workflows.

During the experiment all data generated by the lab is stored directly in the database of the DeviceLayer. From there it is instantly accessible for the process management system (for example to determine the remaining runtime of a device or intermediate measurement results, etc.) but is also archived for later use. When a run of the workflow is started, the lab server generates an ID that ties all pieces of information and results generated in this run together.

The REST‐API of the DeviceLayer can also be used to feed experimental data into an electronic laboratory notebook (i.e. by using an appropriate middleware that “speaks” both APIs) or any other data analysis tool for further processing.

## 4 CONCLUDING REMARKS

In this report, a detailed concept for laboratory digitization is presented. The methodology used focuses on micro‐services, which have clearly defined purposes and interfaces. It thus allows easy maintenance and extension. The data flow is modeled in a strategic way, which ensures data validity and enables easy validation and certification of processes. In contrast to other approaches, which were focused on fully automated applications, the method presented aims at a broad digital integration and flawless interaction with the human worker in the laboratory.

Wherever possible, existing technologies, protocols and standards were used. However, this often raises the need for integration of commercial components with specific communication protocols. In the future, when more and more digital interaction with lab devices will be required, the demand for a vendor independent common communication protocol will grow. For implementing standards like the FAIR‐data principles, an easy method of data acquisition and processing is without alternatives. This is enforced for example by the German NFDI and systems as the one presented in this article will increase data quality and reduce the need for potentially error‐prone manual data processing by the researcher.

Especially in research or quality testing, were hand‐written protocols that are carried out by trained professionals are the standard workflow, a sophisticated method for interaction of the digitized lab with the human worker is necessary. The approach presented focuses on good adoption through easy to use hardware and software that does not interfere with the normal lab process. A great improvement over traditional methods are the possibilities for fully automated documenting and archiving. As all data generated in the lab process are available in one place, creation of analysis protocols can be automated to exploit the full potential of the data.

## CONFLICT OF INTEREST

The authors have declared no conflict of interest.

## Supporting information

Supporting Information

## Data Availability

The data that support the findings of this study are available from the corresponding author upon reasonable request.

## Footnotes

1 The open source SiLA2 standard definition documents can be found online. Part A: https://drive.google.com/file/d/1QWrSD4-YBMwT9HTBzJe3TlBbJSGqq_LC/view Part B: https://drive.google.com/file/d/1a9XJnQUHysW6DQGz4j2m1zngLX-pakxo/view Part C: https://drive.google.com/file/d/1dqQTqRN6vyJy6KBISCsnV_qte_kNh62l/view

2 Both the official reference implementations in several programming languages and also the sila_tecan C# implementation used in this article can be found at: https://gitlab.com/SiLA2

3 The term “RESTful” describes an API that follows the principles of Representational State Transfer (REST), which denotes a HTTP‐based web API that offers information in a structured way (usually in the format of JavaScript Object Notation (JSON) strings) at pre‐defined “endpoints” or URLs (Uniform Resource Locators). The endpoints itself are constant in the server and need to be known *a priori* to the client.

4 Implementation details of this generic approach to user interaction and the principles it implies on digital laboratory protocols are described in the supplementary materials.

5 The mechanisms of abstraction that are used to distribute device runtime information to all UI and its implications on protocol design are discussed in the supplementary material.
