# Supplementary material for: Implementing a digital infrastructure for the lab using a central laboratory server and the SiLA2 communication standard
Source: Eng Life Sci. 2020 Dec 9;21(3-4):208–19. doi: 10.1002/elsc.202000053 (PMC7923558; doi:10.1002/elsc.202000053)
Supplement: Supplementary file 1 — Supporting Information [file ELSC-21-208-s001.pdf]

# Supplementary Information for the Article

***"Implementing a Digital Infrastructure for the Lab Using a Central Laboratory Server and the SiLA2 Communication Standard"***

Marc Porr, Ferdinand Lange, Daniel Marquard, Laura Niemeyer, Patrick Lindner, Thomas Scheper, Sascha Beutel

## A – Process Management in the Digitized Lab

Methods or protocols are "run on the lab" by a process management tool. This connects to the REST-API of the laboratory server, runs commands and interprets results. The DeviceLayer is agnostic of the process management tool used, so in principle any method desired to connect a protocol manager (if necessary by utilizing an appropriate middleware) can be used.

In the solution presented, a process management tool that specifically targets good interaction of the digitized laboratory with the human researches was developed, especially to be used in context with the DeviceLayer.

With this tool standard operation procedures (SOPs) are defined in an easy to learn script-like format. Steps and transitions are defined and calculations or conditions can be implemented in a flexible way. This layout opens up the possibility to extend the infrastructure by a graphical tool for process design later on.

### A.1 – Implementation of the Process Management Tool (ControlFlow)

The process management tool, called "ControlFlow", interacts with the whole lab in one place via the Lab REST API of the DeviceLayer. The ControlFlow runtime can run on any user's computer in the network. It sends command requests to the DeviceLayer and receives result data and device state information from it.

Figure 1 visualizes the architecture of the ControlFlow runtime in context with specific procedures. Procedures and the steps they contain are interpreted by the runtime and all device commands are cached in a command storage. Corresponding calls to the Lab REST API of the DeviceLayer are send and every change of state or (intermediate) result is cached back to the command storage. Timers are running locally in the runtime. A GUI component can be used by the worker in the lab to view the protocol and the current state of step execution.

ControlFlow is implemented as a library in the D programming language. For protocol design, this library offers convenience functions to wrap up calls the DeviceLayer. Protocols are written in a mixin format, so they essentially form "compile time plugins" for the ControlFlow main program. This program presents the lab user with a graphical user interface (GUI) and mechanisms of controlling a running protocol.

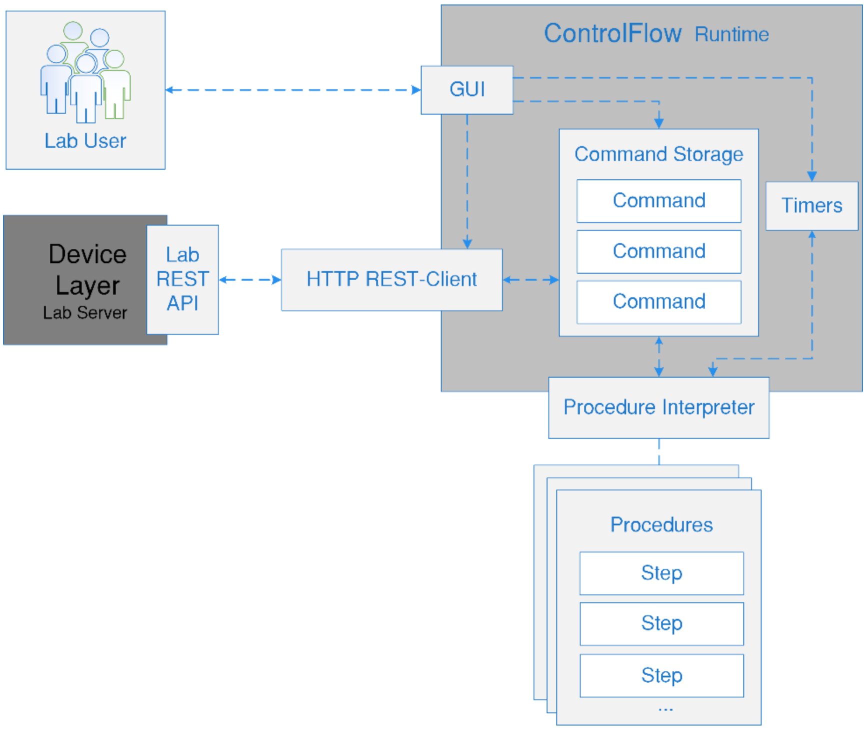


*Figure 1: Architectural overview of the ControlFlow implementation.*

The protocol mixins are compiled together with the main component to a ControlFlow Runtime. This executable offers all protocols that were beforehand defined. This method ensures that somebody executing a protocol must not necessarily have the permissions to alter its steps. Compiling protocols into the executable offers precise control over user rights. The protocol designer (lab manager) can pre-define the steps and this can only be changed by the lab user, when he/she is given the corresponding source code.

To introduce a precisely defined level of flexibility, a parameter file bases approach is taken. Parameters and their boundaries are defines in the protocol itself (and are thus unchangeable in the compiled protocol). However, specific parameter values are defined in a parameter file in JavaScript Object Notation (JSON) format. This file can be changed by the user before executing a protocol. This enables the executor to alter all parameters, which were allowed to be changed by the manager, in pre-defined boundaries. This method ensures that the lab manager has full flexibility during design time, whereas the liberty of adjustment and configuration by the worker in the lab can be specifically fine-tuned.

## A.2 – Protocol Design

Protocols are expressed in the form of scripts that consist of steps and transitions between them. Steps usually contain lab device interaction and/or user interaction via the generic user interface (UI) controller. Transitions specify how the current step can be left for the next one. This can either mean a direct transition with no conditions (devices can thus perform tasks in the background), waiting for a device command or timer to finish, or waiting for some user input – like the confirmation of manual

steps (i.e. pipetting, transferring containers or liquids, etc.). To model loops or forks in the control flow, steps can specify the following step based on conditions or logic operations.

As the “script language” for protocol design is developed as a library for the D programming language, all kinds of complex operations (like mathematical calculations, data operations, etc.) can be performed in a protocol. This flexibility ensures that even complicated workflows can be accurately expressed in one place with all process logic combined together. During the planning-phase of an experiment, the lab manager designs and parametrizes the workflow. This protocol can then be evaluated, validated and probably certified. Later on, every worker in the lab can execute the protocol. The workflow can contain gatekeepers, which only allow the worker to proceed, when certain safety or quality criteria are met. For example in a stirring step, the digitized laboratory can make sure that the duration and stirring speed was sufficient by a defined margin of tolerance. The server generates unique identifiers for every resource, which can be used by the ControlFlow runtime to recall all results and measurements any time in the future.

Figure 2 shows an example protocol that can be executed in the digitized lab. The ControlFlow library is used to specify a simple protocol that consists of three steps. Firstly, a storage object is initialized, that hold information about the protocol and metadata about the UI-devices supported.

The first step displays information for the worker on any UI-device connected and waits for a manual confirmation by the worker. The second step starts a centrifuge with 2000 RPM at 20 C° for 60 seconds and waits for the centrifuge to finish this command. Lastly, a timer is set to 25 seconds and is awaited.

```
module usecases.example;
import controlflow.usecase;
mixin RegisterAllUseCases;

@UseCase void example()
{
    flow.initStorage([UIDev.glass], "TCI", "example", "layouts/example");

    flow.step("prepare", {
        sendLayout("prepareCentrifugeUI");
        uiEvents("next").wait();
    });

    flow.step("centrifuge", {
        auto cmd = command("centrifuge", "Start", 2000, 60, 20);
        cmd.wait();
    });

    flow.step("timer", {
        sendLayout("timerUI");
        wait(timer("wait", 25.seconds));
    });
}
```

*Figure 2: Example protocol file.*

In a protocol, several steps are specified. A step is implemented as a function delegate that can contain any valid D source code. The ControlFlow library offers functionality for device interaction by wrapping the client for the DeviceLayer's Lab REST API. In addition, an abstraction layer for parameters and a generic interface to UI-device interaction methods is available to the protocol designer. When a step delegate is finished, the concurring step is automatically entered by the runtime. This transition can be delayed by waiting for certain tasks. For example, the step can wait for a timer or for a device command to finish. This is necessary, when the command's results are used in the next step. For manual steps, where no device is involved, waiting for an event trigger from an UI-device is necessary. For complex control logic, conditional jumps between steps can be used. Thus, forks, loops and conditional execution of steps can be modeled.

### A.3 – Protocol Execution

After a protocol is packed as an executable ControlFlow Runtime, it can be executed on any user computer that has network access to the laboratory server. When starting the compiled ControlFlow executable with a protocol, the user is presented with a graphical user interface (GUI), which allows managing the protocol's steps and visualizes the data transferred between the devices, the lab server and the process management tool (see Figure 3). On the top right all steps in the protocol can be seen and control flow can be manipulated (repeat steps, jump to steps, pause execution, etc.). On the top left calls to the Lab REST API are listed. For debugging purposes payload can be seen and resending of commands is possible. On the bottom, execution logs from the protocol runtime mechanism are printed to inform the user or lab manager of the precise interaction of the process management with the lab server.

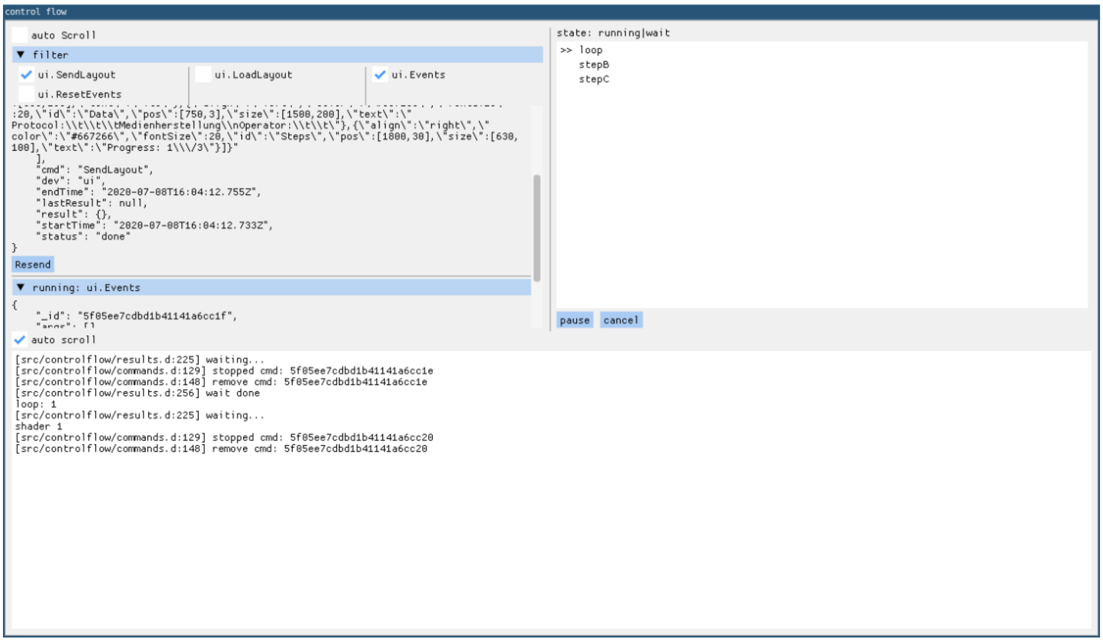


*Figure 3: Screenshot of the ControlFlow GUI during execution of a simple dummy protocol.*

**B – Guiding and Aiding the User during Protocol Execution**

For communication with the worker during protocol execution, different user interface devices (UI-devices) were integrated. These devices offer guidance to the worker in form of short summaries of the current protocol step, safety information or relevant data about the chemical/organism/material worked with. This can also include the results of automatic calculations. For example, when a cell density measurement is performed, the result value is automatically processed with the calibration stored in the protocol and the worker is presented with the resulting cell concentration directly.

The communication of the laboratory system with the researcher during the procedure is modeled using a standard gateway to all possible UI-devices. Thus, a procedure can be agnostic of the UI-device used. It only needs to specify what needs to be presented to the user, not how or by which channel the communication to an end-device is carried out. All used UI devices are connected to the lab server in a standardized way and receive the information necessary to equip the worker with all information specified in the protocol.

In the presented solution, user interaction with two different kinds of UI-devices is implemented. One is based on a consumer-grade tablet that is carried by the worker and placed on the lab bench when a protocol is carried out. The tablet presents visual information and offers buttons for protocol navigation. The other approach uses smart-glasses that are worn by the worker on top of the lab safety glasses. They are a head-mounted display that shows small snippets of visual information right in the view of the worker. Controlling the protocol execution is achieved in this setting by simple voice commands like "confirm", "previous", etc. However, due to the generic nature of the mechanism, any other device can be plugged into the architecture without changing the principles of data flow or the protocols.

**B.1 – User Interaction Abstracted by a Generic User Interface Device**

Communication with different UI-devices is abstracted by a generic UI controller. It is implemented in the same way as any other SiLA2 server. The uniform and generic SiLA2 interface of this controller is consumed by the DeviceLayer and it thus offers the UI functionality in the same way as it is done with any other device's commands by the REST API for the process management tool. This generic device can be used during protocol design. Depending on the UI-devices connected during protocol execution, the content is formatted according to the specific target device.

Figure 4 shows the architecture for integrating UI-devices into the digitized laboratory. Every target device has to run a small REST server that hosts an API, which supports receiving of layout information for display purposes and sending of events. This, for example, is done with an Android app on the tablets and smart-glasses. Other UI-devices can be integrated the same way, as long as the common UI REST API is shared. The generic UI controller connects to all of these UI REST servers of all UI-devices present.

Whenever new information is announced by the ControlFlow runtime, this information (in JSON format) is pre-processed by the UI controller and is sent to all UI-devices connected.

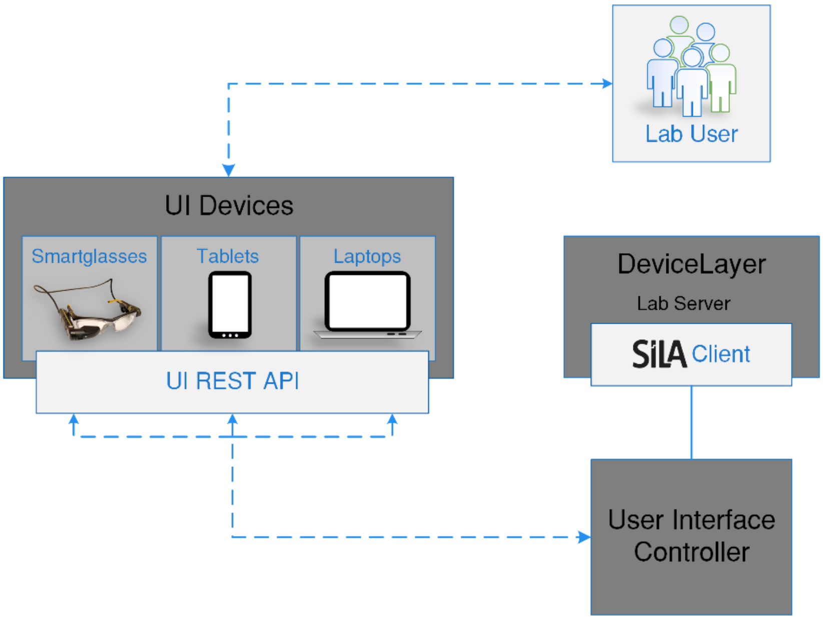


*Figure 4: Architecture of UI-device integration via the generic user interface controller (SiLA2 server).*

When a user interaction event (such as the trigger for a manual confirmation of a step using speech commands or by pressing buttons on the UI-device) is registered on one of the connected UI-devices, this information is passed on via the UI REST API to the generic UI controller. The controller sends it via the DeviceLayer REST API to the ControlFlow runtime. There it is automatically processed and the runtime decides, whether this event is further processed at the current protocol execution state. If, for example, the runtime is waiting for the user to manually confirm a transfer step, the following step is entered after the “confirm”-event was registered by one of the connected UI-devices.

**B.2 – Flexible Layout Files Specify Content**

The process management tool uses JSON data, which is send via the REST API of the DeviceLayer to the generic UI controller, to specify the information that should be presented to the researcher. The Apps running on the specific UI devices interpret the JSON content and generate corresponding UIs. This means, that every device can for example calculate image sizes or text box positions based on its available screen size.

To simplify protocol design, templates for common visual elements are stored in JSON files and are loaded by the ControlFlow runtime during execution. These files can contain variables, which can be filled in by using a variable substitution mechanism in the ControlFlow library. This method ensures that only minimal redundant UI specification is needed inside the protocols.

To display life measurement data, placeholder fields are specified inside the JSON layouts. Every time a new (intermediate) result is generated inside the lab, the DeviceLayer sends an update to the generic UI device controller. It then passes on this information to all connected UI devices and the Apps on these devices can update visual information wherever the new updated field is displayed. In this mechanism, the source devices of results are not described by their specific (unique) IDs, but by their

class. This makes devices interchangeable (for example swapping a scale from manufacturer A for one of manufacturer B) without changing the protocols or layout files.

**B.3 – Example**

Figure 5 displays an example screen, shown to the worker on the smart glasses during a weighing protocol step that is part of a media composition protocol for a bioreactor. The view is updated with the current scale reading in real time during the weighting process. It also shows the desired target weight. The information is displayed right in the field of view of the lab worker, who can work with both hands. The protocol execution waits as long as it takes to weigh in the target weight defined in the protocol by a specific margin of tolerance. This makes sure that no mistake can be made in this step. The exact amount of material is stored in the database after the step is finished and can be used for precise calculation of concentrations or for documentation purposes.

Figure 6 shows the ControlFlow script needed for this example step. The layout template is stored under the name “4_Weight” and contains a variable field named “chemical”. To make use of this template, first the variable “chemical” has to be set to the name of the material used in this step (“yeast exract”) using the “flow.storage.set”-command in line 2. With the “sendLayout”-command in line 3the template is loaded and send to the generic UI controller, which results in the generation of the screen displayed in Figure 5.

Line 4 contains the device command to the scale. It calls the SiLA2 command “WeightTotargetValue” which needs two parameters. The first is the target Value (5g in this case) and the second is the margin of tolerance that is considered acceptable. The “waitUntilDone” command acts as a gatekeeper and makes sure that the step can only be left for the following one, when the weighting command reports a mass inside the defined range.

The used layout template “4_Weight” is shown in Figure 7. It describes the format of the information to display in a JSON structure. The “mode” field describes the type of events that the UI device needs to listen. As this step should only be left by performing the weighting task, this is set to “none”.

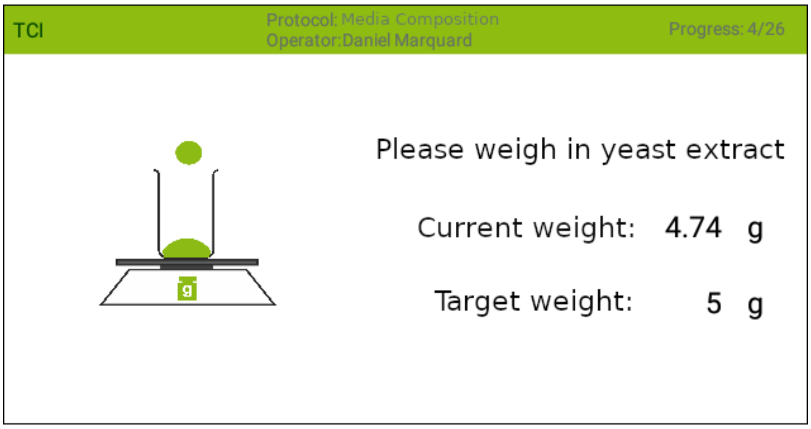


*Figure 5: Example view of the content presented on the smart glasses to the lab worker during a weighting step that is part of a media composition protocol for a bioreactor.*

```
flow.step("Weigh Yeast Extract", {
    flow.storage.set("chemical", "yeast extract");
    sendLayout("4_Weight");
    command("Scale", "WeightToTargetValue", 5, 0.1).waitUntilDone();
});
```

*Figure 6: ControlFlow script for the example step described above*

The layout defines one image and three text boxes. One shows the descriptive text, which also contains the variable “chemical” which is overwritten by the value used in the ControlFlow script. This architecture makes layout templates flexible and reusable and thus minimizes repetitive declarations. The next two text boxes do not contain fixed values. They are linked to SiLA2 result fields of the Scale. When the command to the scale is issued and the “WeightToTargetValue” command reports intermediate results to the DeviceLayer, these fields are dynamically updated.

```
{
    "mode": "none",
    "textBoxes": [
        {
            "pos": [ 45, 30 ],
            "text": "Please weigh in $(chemical)"
        },
        {
            "pos": [ 45, 60 ],
            "id": "Scale:CurrentValue"
        },
        {
            "pos": [ 45, 80 ],
            "id": "Scale:TargetValue"
        }
    ],
    "images": [
        {
            "pos": [ 15, 30 ],
            "img": "picto/BeakerScaleAdd.png"
        }
    ]
}
```

*Figure 7: Simplified example layout template that describes the UI information used to create the UI screen shown in the example above.*

## C – ControlFlow script for Water Analysis Example Workflow

The following Listing contains the ControlFlow script for the PCR based water analysis workflow described in the example section of the article.

```
module usecases.waterAnalysis;

import controlflow.usecase;
import std.json;
import std.file;

mixin RegisterAllUseCases;

@UseCase void waterAnalysis()
{
    // read Parameters from config-file
    JSONValue config = parseJSON(readText("params/waterAnalysis.json"));

    // create UI Helper
    flow.initStorage([UIDev.tablet, UIDev.glass], "TCI", "PCR Water Analysis",
"layouts/waterAnalysis");


    // setup the workflow:

    flow.step("Login with personal QR-code", {
        flow.storage.set("name", "PCR Water Analysis");
        sendLayout("Login");
        registerUser(waitForQrCode());
    });

    flow.step("Heat Thermo Shaker", {
        command("thermoshaker", "Heat", config.opt!int("Temperature"));
    });

    flow.step("Register Digestion Buffer", {
        string chemname = "Digestion Buffer";
        flow.storage.set("chemical", chemname);
        sendLayout("Scan");
        registerChemical("digestion_buffer", chemname);
    });

    flow.step("Pipet Digestion Buffer", {
        flow.storage.set("chemical", "Digestion Buffer");
        flow.storage.set("target", "Reaction Vessel");
        sendLayout("Pipet");
        command("pipet", "SetVolume", config.opt!float("BufferVolume"));
        wait(uiEvents.Confirm);
    });

    flow.step("Pipet Water Sample", {
        flow.storage.set("chemical", "Water Sample");
        flow.storage.set("target", "Reaction Vessel");
        sendLayout("Pipet");
        command("pipet", "SetVolume", config.opt!float("SampleVolume"));
        wait(uiEvents.Confirm);
    });

    flow.step("Transfer to ThermoShaker", {
        flow.storage.set("message", "Place Reaction Vessel in Thermo Shaker");
        sendLayout("Message");
        wait(uiEvents.Confirm);
    });

    flow.step("Digest", {
        flow.storage.set("message", "Please wait for Digestion");
        sendLayout("Wait");
        auto timeout = timer(5.minutes);
        wait(timeout);
    });

    flow.step("Stop Thermo Shaker", {
        command("thermoshaker", "Stop");
    });

```

```
    flow.step("Transfer to Centrifuge", {
        flow.storage.set("message", "Take Reaction Vessel out of Thermo Shaker, place it in Centrifuge. Place Counter Weight.");
        sendLayout("Message");
        wait(uiEvents.Confirm);
    });

    flow.step("Run Centrifuge", {
        sendLayout("CentrifugeRun");
        auto cmd = command("centrifuge", "Run", config.opt!int("CentriRPM"), config.opt!int("CentriRuntime"));
        cmd.waitUntilDone();
    });

    flow.step("Transfer out of Centrifuge", {
        flow.storage.set("message", "Take Reaction Vessel out of Centrifuge");
        sendLayout("Message");
        wait(uiEvents.Confirm);
    });

    flow.step("Register PCR Reagents", {
        string chemname = "PCR Master Mix";
        flow.storage.set("chemical", chemname);
        sendLayout("Scan");
        registerChemical("pcr_mastermix", chemname);
    });

    flow.step("Pipet Sample Supernatent", {
        flow.storage.set("chemical", "Supernatent of Water Sample");
        flow.storage.set("target", "PCR Vessels");
        sendLayout("Pipet");
        command("pipet", "SetVolume", config.opt!float("SupernatantVolume"));
        wait(uiEvents.Confirm);
    });

    flow.step("Pipet PCR Reagents", {
        flow.storage.set("chemical", "PCR Reagents");
        flow.storage.set("target", "PCR Vessels");
        sendLayout("Pipet");
        command("pipet", "SetVolume", config.opt!float("PCRReagentsVolume"));
        wait(uiEvents.Confirm);
    });

    flow.step("Transfer into PCR Cycler", {
        flow.storage.set("message", "Transfer PCR Vessels into PCR Cycler");
        sendLayout("Message");
        wait(uiEvents.Confirm);
    });

    flow.step("Run PCR Cycler", {
        sendLayout("PCRRun");
        auto cmd = command("pcr", "RunProgram", config.opt!int("PCRProgramName"));
        cmd.waitUntilDone();
    });

    flow.step("End", {
        sendLayout("Finish");
    });
}
```
